# Association of *T. gondii* infection with suicide: a systematic review and meta-analysis

**DOI:** 10.1186/s12889-020-08898-w

**Published:** 2020-05-24

**Authors:** Eissa Soleymani, Fariba Faizi, Rashid Heidarimoghadam, Lotfollah Davoodi, Younes Mohammadi

**Affiliations:** 1 Students Research Committee, School of Medicine, Hamadan University of Medical Sciences, Hamadan, Iran; 2 Research Center for Health Sciences, School of Public Health, Hamadan University of Medical Sciences, Hamadan, Iran; 3 Antimicrobial Resistance Research Center, Mazandaran university of Medical science, Sari, Iran; 4 Modeling of Noncommunicable Diseases Research Center, School of Public Health, Hamadan University of Medical Sciences, Hamadan, Iran; 5 Department of Epidemiology, School of Public Health, Hamadan University of Medical Sciences, Hamadan, Iran

**Keywords:** *T. Gondii*, Suicide, Systematic review, Meta-analysis

## Abstract

**Background:**

Findings on the association between Toxoplasma gondii (*T. gondii*) infection and suicide are contradictory. This paper aimed to resolve this uncertainty by conducting a meta-analysis.

**Methods:**

We found the relevant studies using keywords include “Toxoplasmosis” and “Suicide” and the related synonyms in international databases such as ISI, Medline, and Scopus. The eligible studies were included in the meta-analysis phase. The random effect approach was applied to combine the results.

**Results:**

Out Of 150 initial studies, 15 were included in the meta-analysis. Odds of suicide in people with *T. gondii* infection was 43% (OR: 1.43, 95%CI; 1.15 to 1.78) higher than those without this infection. The test for publication bias was not statistically significant, which indicates the absence of likely publication bias.

**Conclusion:**

This study confirms that *T. gondii* infection is a potential risk factor for suicide. To reduce cases of suicide attributable to *T. gondii* infection, it is recommended to implement some measures to prevent and control the transmission of the disease.

## Background

Suicide, as one of the major health threats for humans, leads to more than 800,000 deaths globally each year, such that one person per second dies from suicide. Therefore, suicide accounts for 1.5% of all deaths [1].

Many risk factors increase the risk of suicide. The mental disorders, misuse of drugs, mental states, cultural factors, family, and social and genetic conditions elevate the risk of suicide [2].

*Toxoplasma gondii (T. gondii)* is one of the most common parasites in humans. This parasite exists in approximately one-third of the world’s population and also more than 40 million people in the United States [3, 4]. The life cycle of this parasite occurs in intermediate hosts such as humans (by asexual reproduction) and felines being the definitive hosts for sexual reproduction. Consumption of *T. gondii* -contaminated food, vegetable, water, and muscle cysts present in undercooked meat and also congenital infection is the most common route of disease transmission [5–7]. Infection by this parasite in pregnancy can lead to mental disorders and deafness, abortion, and vision disturbances after birth [8, 9]. This parasite leads to severe complications such as encephalitis and pneumonitis in immunocompromised persons (such as organ recipients and cancer or HIV/AIDS patients). However, in immunocompetent individuals, clinical signs are mild and self-limited such as fever and cervical lymphadenopathy [10–12]. Moreover, latent infections are frequently associated with tissue cyst of *T. gondii* in the skeletal muscle and brain tissue, leading to psychiatric complications [12, 13]. The tachyzoite form of the disease is responsible for the acute stage of the infection [13]. It has been well documented that *T. gondii* infection may lead to changes in the behavior of its hosts [3, 4]. It has been reported that the *T. gondii* infection may cause reduced Intelligence Quotient (IQ) [14], personality changes [15], and psychomotor performance [16]. *T. gondii* infection affects the behavior of humans, such that recent clinical data demonstrate that *T. gondii* infection antibody may play a role in the pathophysiology of suicide. The studies documented that these elevated levels of cytokines are associated with depression and suicide [17].

However, the results of the studies on the association of *T. gondii* infection and suicide are not consistent. While some studies claim that there is no association between suicide and *T. gondii* infection [18, 19], some others suggest that these two factors are correlated [20]. One of the resolutions to overcome this conflict is to perform a meta-analysis, which is a method to extract one single effect size from several multiple studies. If studies can extract a causal association between *T*. *gondii* infection and suicide, we may identify persons with an increased probability of suicide and thus find ways to prevent it.

The present study aimed to provide a summary estimate for the association of *T. gondii* infection with suicide and to evaluate whether *T. gondii* is associated with the risk of suicide or not.

## Methods

Preferred Reporting Items for Systematic Reviews and Meta-Analyses (PRISMA) tool was applied to conduct this study.

### Protocol and registration

The protocol was registered in Hamadan University of Medical Sciences (No. 9710256386).

### Eligibility criteria

Based on Population, Intervention, Comparison, Outcome, Study type (PICOS) principles, we selected the analytical studies (including case-control, cohort, cross-sectional) that reported an association between *T. gondii* infection (as a predictor) and suicide (as outcome) in all age and sex groups of the population. In this process, we did not set any time limitation on the selection of the studies.

### Information sources

Medline, ISI, and Scopus databases were searched to retrieve the related studies up to 25 March 2019. Moreover, we searched the reference list of the screened studies to find the missed studies.

### Searching literature

Two major keywords including suicide (such as Suicide OR Suicides OR ‘Suicide, Attempted’ OR ‘Attempted Suicide’ OR Parasuicide OR Parasuicides OR ‘Suicide, Completed’ OR ‘Completed Suicides’ OR ‘Suicides, Completed’ OR ‘Completed Suicide’) AND *T. gondii* (*Toxoplasma* OR *Toxoplasmas* OR ‘*Toxoplasma gondii*’ OR ‘*Toxoplasma gondius*’ OR ‘*gondius*, *Toxoplasma*’ OR *Toxoplasmosis* OR ‘*Toxoplasma gondii* Infection’ OR ‘Infection, *Toxoplasma gondii*’) were used to construct a search strategy for each database. In Pubmed, we searched for studies using Mesh terms. In Scopus, the search was done on title, abstract, and keyword. In Web of Sciences, the studies were searched based on the topic.

### Study selection

We selected those studies that assess the association of *T. gondii* with a suicide. Two independent reviewers searched the databases and then screened the title, abstract, and full text of the studies to choose the relevant studies. The disagreement between the two reviewers was resolved by a third person.

### Data collection process

An EXCEL sheet was designed to extract the required data of the selected studies. The sheet included the name of the first author, year of publication, country of the study, age, sex, sample size, and effect size of the association.

### Risk of bias in individual studies

Newcastle and Ottawa statement (NOS) checklist was applied to assess the quality of the studies.

### Summary measures

Odds Ratio (OR) with 95% Confidence Interval (95%CI) was determined as the effect size for this study.

### Synthesis of results

The final selected studies were included in the meta-analysis. A random-effect approach was used to combine the studies and produce one single estimate. I^2^ statistics and chi-square tests were used to assess the existence of heterogeneity among the studies.

### Risk of bias across studies

We used Egger and Begg test to investigate publication bias in reporting the results.

## Results

**Fig. 1:**
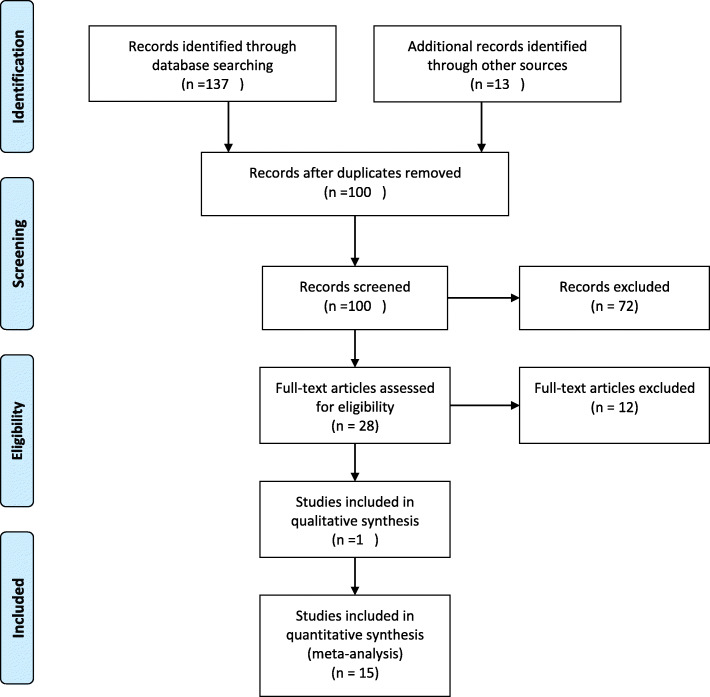
Process of performing the systematic review

Figure 1 demonstrates the process of performing the study. The initial search in the databases yielded 150 studies. After discarding duplicates and irrelevant studies, 15 studies were qualified to be included in the quantitative analysis phase (Meta-analysis), and one study included in the qualitative phase [4, 17–31].

Table 1 shows the characteristics of the studies included in final phase. In terms of study setting, the selected studies were conducted in the United States, Turkey, Germany, Mexico, Poland, Denmark, France, Russia, South Korea, and Iran. Out Of 16 studies, seven reported the positive association of *T. gondii* infection with a suicide, and eight studies did not find any significant relationships between *T. gondii* infection and suicide. By contrary, one study found a protective association between *T. gondii* and suicide.

**Table 1:** Characteristics of the studies included in final phase

Author	Country	age	Male	Female	Design	Sample size	Number of Suicide Case	Positive Cases	Number of Control group	Positive controls	Conclusion	Quality score (out of 8)	Reference
**Arling (2009)**	United states	40±10	90	149	Case-control	234	81	11	153	17	Positive association	8	[18]
**Yagmur F (2010)**	Turkey	24.3±7.6	82	318	Case-control	400	200	82	200	56	Positive association	7	[31]
**Okusaga (2014)**	Germany	38.6±11.1	600	350	Case-control	950	351	146	599	226	No association	8	[19]
**Pedersen, M. G (2012)**	Denmark	Pregnancy ages	–	45,788	prospective cohort study	45,788	N/A	N/A	N/A	N/A	Positive association	6	[29]
**Alvarado-Esquivel, C (2013)**	Mexico	All ages	88	195	Case-control	283	156	7	127	10	Negative association	7	[22]
**Samojlowicz, D (2013)**	Poland	19 to 86 years	115	12	Case-control	127	41	26	86	42	No association	6	[30]
**Coryell W (2016)**	United states	17.5 ± 1.7	30	78	Case-control	108	17	2	91	2	No association	7	[19]
**Gale, S. D (2016)**	United states	20 to 80 years	2469	3018	Cross-Sectional	5487	NA	NA	NA	NA	No association	7	[27]
**Okusaga, O (2016)**	United states	40±11.5	518	307	Case-control	825	308	127	517	200	No association	8	[18]
**Sugden, K (2016)**	United states	3 to 38 years	423	414	prospective cohort study	837	16	8	821	228	No association	8	[20]
**Ansari-Lari, M (2017)**	Iran	40±10	72	27	Case-control	99	29	8	70	34	No association	7	[5]
**Dickerson, F (2017)**	United states	38.6±13	88	74	Case-control	162	N/A	N/A	N/A	N/A	Positive association	8	[25]
**Bak, J (2018)**	South Korea	43.75 ± 16.75	141	149	Case-control	290	155	21	135	8	Positive association	7	[23]
**Dickerson, F (2018)**	United states	36±12	647	645	prospective cohort study	1292	N/A	NA	N/A	NA	Positive association	8	[24]
**Fond, G (2018)**	France	32±8.6	184	66	prospective cohort study	250	9	7	241	177	No association	8	[26]
**Ling, Vinita**	Eurepe	0–75+	–	–	Ecologcal study	–	–	–	–	–	Positive association	7	[32]

The strongest and the weakest associations were reported by Dickerson (2.41) and Okusaga (1.14), respectively.

**Fig. 2:**
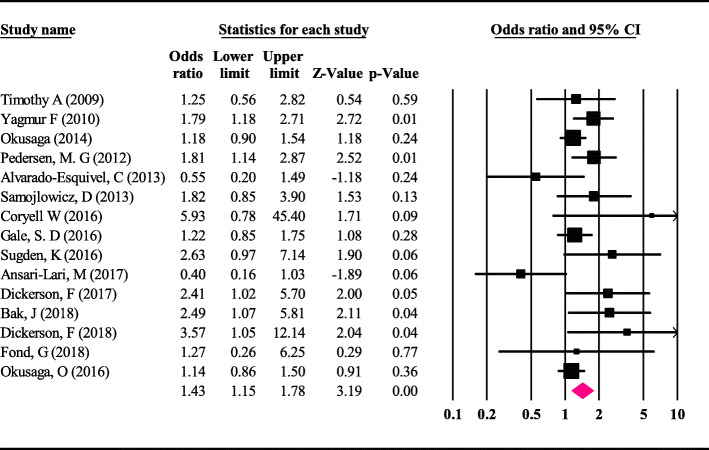
Odds ratio with 95% confidence interval for the studies and meta-analysis

The results of the meta-analysis indicates that the odds ratio of *T. gondii* infection and suicide was 1.43 (95%CI; 1.15 to 1.78), which is statistically significant (Fig. 2). Therefore, it can be stated that a person is seropositive in terms of *T. gondii* if has a 43% risk of suicide compared with the non-infected person.

The Begg test was not statistically significant (*P*-value = 0.28) in assessing the existence of publication bias, indicating the absence of publication bias in the study.

Examining the degree of heterogeneity among studies using the I^2^ test demonstrated a moderate heterogeneity (I^2^ = 0.71). Therefore, we used a random-effect model to perform the meta-analysis. In addition, Beg’s test was not significant, which indicate absence of publication bias (*P* = 0.26).

In the qualitative phase, one study assessed the association of *T. gondii* infection with suicide using ecological studies. This study showed that after adjusting potential confounders, there is a significant association between seroprevalence of *T. gondii* infection and suicide rate among European countries [31].

## Discussion

In this paper, we presented the result of meta-analysis for the association of *T. gondii* infection and suicide. Combining the results of 15 eligible studies, we confirmed that developing *T. gondii* infection may increase the risk of suicide by 43%. Therefore, individuals with *T. gondii* infection had higher probability to suicide than without *T. gondii* infection. Although, strength of the obtained risk in our study is not very substantial, it is a remarkable risk should be considered.

The literature has been provided the evidence for existence of association of *T. gondii* infection with mood disorders such as schizophrenia [32], bipolar disorder [33, 34] and suicide [35]. In a review paper published by Pao-Chu Hsu, it was concluded that *T. gondii* is associated with mental health disorders such as schizophrenia, suicide attempt, depression, and other neuropsychiatric diseases [36]. Moreover, in an ecological study investigated association of *T. gondii* with Suicide rates in women, it revealed that there is a positive association between rates of infection with *T. gondii* and suicide in 20 European countries and suicide is more common in women of postmenopausal age [31] .

The studies explained the mechanism of association of *T. gondii* infection with behavioral changes well. After proliferation of this protozoan parasite in different organs during the acute phase, the parasite preferentially forms cysts in the brain and establishes a chronic infection that is a balance among parasite’s evasion of the immune response and host immunity. Different cells of brain, such as neurons and astrocytes, can be infected. In laboratory surveys using non-brain cells have showed deep effects of the infection on gene expression of host cells, containing molecules that increase the immune response and those involved in signal transduction pathways, suggesting that similar effects could happen in infected cells of brain. *T. gondii* infection also appears to affect signaling pathways in the brain. Consequently, chronic infection reactivation with the parasite (rupture of cyst and proliferation of tachyzoites) in the brain may play a role in the onset of the disease [37]. In fact, *T. gondii* act on suicide behavioral through two pathways: disturbance in dopamine synthesis and activation of indoleamine-2,3-dioxygenase (IDO) that reduce amount of serotonin in brain [36]. The studies show that individuals who suicide had a significantly higher IgG antibody to *T. gondii* compared with those without a suicide [17]. Moreover, a study showed the association of *T. gondii* antibodies and suicidal behavior in patients with schizophrenia, which is consistent with reports on associations between *T. gondii* and suicidal behavior in patients with mood disorders [17], overall psychiatric patients [20, 31]. *T. gondii* infection plays a role in the higher later occurrence of suicide in lifespan [31]. Experimental studies have shown that the relationship between *T. gondii* infection and suicide is reinforced by the relative tropism of *T. gondii* cysts in greater density in the amygdala nucleus or the frontal cortex, which are normally involved in regulating behavior [38]. The following explanations can illuminate the probable mechanisms of a relationship between *T. gondii* and suicidal behavior. First, *T. gondii* induces the production of pro-inflammatory cytokines (IFN- γ, IL-6, and IL-12) by activating lymphocytes and macrophages [39]. Interferon-gamma, by triggering lymphocytes and macrophages, blocks the development of *T. gondii* [40]. In response to the *T. gondii*, cytokines are produced, leading to an increase in the activity of enzymes kynurenine monooxygenase (KMO) and indoleamine 2, 3-dioxygenase (IDO). In the metabolism of the amino acid tryptophan, KMO and IDO are restricted. Tryptophan evacuation via the kynurenine pathway (3-hydroxyl kynurenine, 3-OH-kynurenine, and quinolinic acid, QUIN) limits the growth and spread of infection [39]. Moreover, it can decrease neurotransmitter serotonin synthesis in the brain and may raise the susceptibility to triggering suicide risk factors such as depression, impulsivity, and aggression [41]. Changes in glutamate and dopamine neurotransmission have shown a key role in suicide and suicidal behavior [42, 43]. Finally, changes in neurotransmitters can play a role in behavioral development that increases the risk of suicide [35, 44].

In this study, we had several limitations. One major limitation of the present study is that we just included English language studies and overlooked non-English ones. Therefore, we cannot assess the effect of non-English studies on our results. Second, we included only studies had full text, and therefore, we excluded the studies without full text. However, it seems that more investigations on the association of *T. gondii* infection with suicide, especially on mechanisms of pathogenesis of *T. gondii* infection in suicidal behavior in required. Furthermore, updating the review articles about *T. gondii* infection and suicidal behavior without time and language limitations is suggested.

Despite the mentioned limitation, this study provides important clues to inform policy-makers about the serious role of *T. gondii* infection in a suicide. Therefore, considering the consequences and complications of *T. gondii* infection such as suicide, control, prevention, and its treatment, this parasitic infection must be highly considered.

## Conclusion

Our study is the first meta-analysis and systematic review to assess the association of *T. gondii* infection and a suicide. *T. gondii* significantly increases the risk of a suicide. Therefore, to reduce the risk of the suicide associated with *T. gondii*, it is recommended to take some measures to prevent and control of transmission of T. gondii.

## Data Availability

The corresponding author is responsible for data. Access to all relevant raw data will be free to any scientist.

## Abbreviations

IQ: Intelligence Quotient
PRISMA: Preferred Reporting Items for Systematic Reviews and Meta-Analyses
OR: Odds Ratio
CI: Confidence Interval
NOS: Newcastle and Ottawa statement
